# Longitudinal analysis of short-chain fatty acid profiles in stool of sleeve gastrectomy patients

**DOI:** 10.1038/s41387-026-00441-x

**Published:** 2026-06-11

**Authors:** Oliwia Lange-Andrzejewska, Aleksandra Budny, Agata Janczy, Maciej Wilczynski, Michal Szymanski, Monika Proczko-Stepaniak, Tomasz Sledzinski, Adriana Mika

**Affiliations:** 1 Department of Environmental Analysis, University of Gdansk, Gdansk, Poland; 2 Department of Hypertension & Diabetology, Faculty of Medicine, Medical University of Gdansk, Gdansk, Poland; 3 Division of Food Commodity Science, Faculty of Health Sciences with the Institute of Maritime and Tropical Medicine, Medical University of Gdansk, Gdansk, Poland; 4 Department of General, Endocrine, and Transplant Surgery, Faculty of Medicine, Medical University of Gdansk, Gdansk, Poland; 5 Department of Pharmaceutical Biochemistry, Medical University of Gdansk, Gdansk, Poland

**Keywords:** Lipidomics, Obesity, Nutrition, Fatty acids

## Abstract

Obesity presents significant health risks, including metabolic disorders and depressive symptoms, necessitating effective interventions such as sleeve gastrectomy (SG), which enables substantial weight loss and metabolic improvements. The primary question of this study was whether SG also affects faecal short-chain fatty acid (SCFA) profiles over 12 months, and whether these changes are related to anthropometric, biochemical, and psychological parameters. A total of 37 female patients with obesity were included in this prospective, observational study. Patients underwent SG and were followed for 12 months postoperatively. SCFA profiles were analysed by gas chromatography-mass spectrometry (GC-MS). Faecal samples for SCFA analysis, anthropometric measurements, blood biochemical markers, food intake and psychological assessments were collected at baseline and at regular intervals after surgery. Our results indicate that SG leads to significant reductions in body mass index, lipid profiles, and systemic inflammation markers, with concurrent alterations in SCFA concentrations, particularly a decrease in major SCFAs (acetic, propionic, and butyric acids) over time. An increase in branched SCFAs was observed post-surgery, which may reflect shifts in the gut microbiota composition and fermentation processes. Patients also reported improvements in emotional well-being and dietary habits. These findings support the hypothesis that SG induces changes in gut microbiota metabolism and underscore the complex interplay between bariatric surgery, gut microbiota, SCFA metabolism, and psychological health. They highlight the need for further research to clarify the long-term implications of these changes and the mechanisms involved.

## Introduction

Obesity is a serious global health problem, and its prevalence continues to rise. This trend incurs high economic and health costs, including increased risk of diseases such as diabetes, hypertension, dyslipidaemia, sleep apnoea, osteoarthritis, and various types of cancer [1]. Obesity has also been associated with depressive disorders [2]. Recent reviews have emphasised the bidirectional relationship between these two conditions, which, when combined, may significantly diminish patients’ quality of life and contribute to social disability [3]. It is predicted that over 1.12 billion people will be struggling with obesity by 2030 [4]. Obesity is a severe condition that must be treated. Bariatric surgery (BS) is the most effective treatment option for patients with a body mass index (BMI) ≥ 35. Sleeve gastrectomy (SG) is the most frequently performed BS procedure due to its relative simplicity and lower complication rates. SG enables patients to lose approximately 30% of their total body weight within a year [5], which also alters the host gut microbiota [6] and subsequently affects the levels of microbiota-derived metabolites, including short-chain fatty acids (SCFAs). SCFAs, which are produced in significant amounts through intestinal fermentation, play an important role in maintaining the integrity and impermeability of the epithelial barrier. Additionally, they act as signalling molecules that influence inflammation, glucose and lipid metabolism, appetite regulation, and energy expenditure. SCFAs prevent the development of obesity by stimulating the secretion of hormones such as peptide YY and glucagon-like peptide-1 (GLP-1), which regulate appetite, food intake, and body weight [7]. Interestingly, overweight individuals and patients with obesity exhibit higher total faecal SCFA concentrations than lean individuals, possibly due to shifts in gut microbiota or increased microbial production [8]. Research also suggests that G-protein coupled receptors such as GPR41 or GPR109a, which bind SCFAs, are present in various regions of the brain, where they exhibit anti-anxiety and anti-depressant effects, while also promoting memory enhancement and neuroplasticity [9]. Faecal propionate (C3) levels were lower, whereas 4-methylpentanoic acid (4-M-C5) levels were higher in female patients with depression. Additionally, a strong correlation was identified between acetate (C2), C3, and cognitive as well as somatic symptoms [10]. As previously mentioned, SG leads to alterations in the gut microbiota composition. However, the long-term effects of SG on faecal SCFAs have not yet been fully described. Previous studies in SG patients have suggested that within 4–6 weeks after the procedure, the total faecal SCFA concentration, along with C2, C3, and butyrate (C4) concentrations, decreases [11]. Furthermore, another study reported a reduction in C2, C3, and C4 in the faeces of patients at 3 and 6 months after SG. 6 months after the procedure, both total SCFA and total major SCFA levels were reduced. Additionally, changes in faecal SCFA concentrations following SG were positively correlated with markers such as serum triglycerides (TG) and total cholesterol (T-CH) [12]. In the aforementioned studies, the authors did not consider the patients’ dietary habits. Furthermore, their analysis was limited to a follow-up period of no more than 6 months post-procedure. Thus, a more complex study on the effect of SG on faecal SCFA concentrations is needed.

The aim of our study was to compare faecal SCFA profiles in female patients with obesity before and at 3, 6, 9, and 12 months after SG. We also compared these profiles with anthropometric parameters and serum biomarkers such as lipid profile, glucose homoeostasis markers, and C-reactive protein (CRP). Moreover, we assessed eating habits and mental health by collecting appropriate questionnaires from patients. Taken together, our results show the association between SCFA changes after SG and the metabolic health of patients.

## Subjects and methods

The study group consisted of 37 female patients with obesity (mean age 42 ± 9.3 years) who underwent SG at the Centre of Obesity and Metabolic Diseases of the Medical University of Gdansk. To achieve greater homogeneity of the study population, only female patients were included in the analysis, as they represent the predominant group among individuals undergoing BS. The study was conducted in accordance with the Declaration of Helsinki, and the Medical University of Gdansk Ethics Committee approved the study protocol (no. NKBBN/495/2022). All participants provided written informed consent before participation. The exclusion criteria were age under 18 or over 65 years, previous BS, neurodegenerative diseases, specific or non-specific bowel diseases, recurrent diarrhoea, unusual dietary habits, prolonged stays abroad, history of microbiota transplantation, current probiotic therapy, long-term antibiotic therapy or antibiotic use within 3 months before inclusion in the study. All patients received the same dietary advice before and after the surgery, following a two-stage nutritional intervention. First, a conventional hypocaloric diet was introduced to gradually reduce weight and optimise metabolism. In the immediate preoperative phase (usually 2 weeks prior to surgery), patients followed a ‘liver-shrinking diet’ characterised by a significant reduction in carbohydrate intake with adequate protein and fat intake to promote rapid liver volume reduction and visceral fat loss. This approach aims to improve surgical access and reduce intraoperative risks. In the first week after SG, the patients followed a liquid diet, which was then changed to a semi-liquid and mushy diet for the following 2 weeks. Thereafter, the solid food was gradually reintroduced, with full nutritional intake usually achieved in the 4th postoperative week. Following this period, the patients were advised to adhere to specific dietary guidelines to support long-term weight management and avoid nutritional deficiencies. These recommendations include the consumption of protein-rich meals to maintain muscle mass, portion control to account for reduced gastric capacity, adequate fluid intake, and avoidance of high-calorie and carbonated beverages. In addition, lifelong supplementation of essential vitamins and minerals, including vitamin B12, iron, calcium, and vitamin D, is necessary to reduce the risk of deficiency symptoms.

### Sample collection

**Fig. 1:**
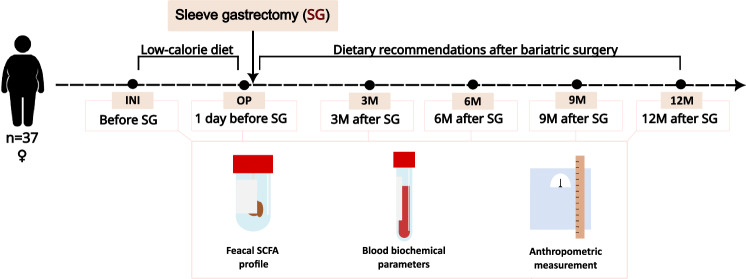
The diagram of the study flow. INI – initial timepoint, OP – operation timepoint.

Stool samples were collected at home using a specimen collection system. Patients were instructed to collect samples the day before their pre-surgery visit (INI), the day before the planned surgery (OP), and the day before their follow-up appointments at 3, 6, 9, and 12 months after surgery. The study flow is presented in Fig. 1.

Samples were frozen for storage, transferred to the laboratory, and stored at −80 °C until analysis. Furthermore, fasting blood samples were collected before the procedure and at each follow-up appointment. Laboratory parameters were determined at the Central Clinical Laboratory of the Medical University of Gdansk. Eating habits were assessed at each time point using 3-day food diaries. Nutritional intake was analyzed in the Nuvero application (Nuvero – Voix, Koszalin, Poland). Data regarding mental state were collected using the Beck Depression Inventory (BDI-II) and Eating Attitudes Test (EAT-26) questionnaires [13, 14]. The EAT-26 was not administered preoperatively due to the ongoing pre-surgical low-calorie diet, which could have biased results by reflecting temporary, goal-directed eating behaviours rather than the actual disordered eating risk.

### Sample preparation

The deuterated internal standard (IS) stock - hexanoic acid-6,6,6-d3 (Cayman Chemical, Ann Arbor, MI, USA) was diluted with ethanol to obtain a 10 µg/ml solution. The samples were prepared in glass tubes. An aliquot of faeces (50 mg) was mixed with 500 µl of Mili-Q water (Hydrolab, Poland) and 25 µl of IS, acidified to pH 2, vortexed and held in an ultrasound bath for 5 min. Then, the samples were centrifuged at 6000 RPM for 5 min. The supernatant was transferred to a clean tube, and 500 µl of diethyl ether (DE) was added. After centrifugation under the same conditions, the upper DE layer was transferred to a glass vial. This extraction step was repeated three times. Subsequently, 40 µl of N-methyl-N-(trimethylsilyl)trifluoroacetamide (MSTFA) (Merck, Darmstadt, Germany) was added for trimethylsilyl (TMS) derivatization, and the samples were incubated at 70 °C for 20 min. Once they reached room temperature, the samples were ready for gas chromatography-mass spectrometry (GC-MS) analysis. All solvents used in the study were of analytical grade and purchased from POCH (Gliwice, Poland).

The analysis of TMS-derivatives was performed using GCMS-QP2020 NX (Shimadzu, Kyoto, Japan) with a Zebron ZB-5MSi column of 30 m × 0.25 µm i.d. x 0.25 µm film thickness (Phenomenex, Torrance, CA, USA). The GC oven temperature started at 40 °C for 2 min, then increased at a rate of 10 °C/min to 70 °C (held for 1 min), then increased at 10 °C/min to 179 °C, and finally increased at 40 °C/min to 300 °C. The total run time was 20 min. The ion source and interface temperatures were set to 230 °C and 250 °C, respectively. The detector was turned on 3 min after injection. The column head pressure was maintained at 100 kPa with helium as the carrier gas. Ionisation was performed at an electron energy of 70 eV in SIM mode (Supplementary Table S1).

### Statistics

All statistical calculations were performed using SigmaPlot 14.5 (Systat Software Inc., San Jose, CA, USA). Comparisons between time points were made using Repeated Measures Analysis of Variance (RM ANOVA) followed by *post-hoc* tests. Results from INI, 3 M, 6 M, 9 M and 12 M time points were compared to the OP time point. Graphs were prepared using Prism 8.0.1 (GraphPad Software, Boston, MA, USA). Spearman’s rank correlation was used to calculate coefficients between variables. The significance level was set at *p* < 0.05.

## Results

### Biochemical and anthropometric parameters

Routine blood and anthropometric parameters obtained at the studied time points are summarised in Table 1. At baseline, patients were characterised by a high BMI as well as T-CH, LDL cholesterol (LDL-CH) and CRP concentrations, with mean values exceeding reference ranges. Following the SG procedure, there was a significant reduction was observed in BMI, LDL-CH, TG, CRP, insulin, glucose, glycated haemoglobin (HbA1c), and Homoeostasis Model Assessment-Insulin Resistance (HOMA-IR) levels. Conversely, HDL cholesterol concentration increased significantly. These results indicate a marked improvement in lipid and glucose metabolism as well as reduced inflammation after SG. The surgical intervention led to a significant and continuous weight loss; both body weight and BMI decreased progressively throughout the 12-month follow-up, reflected by a significant increase in the percentage of total weight loss (%TWL) at each studied time point (*p* < 0.001; Table 1).

**Table 1:** Biochemical and anthropometric characteristics of the study subjects (mean ± SD).

*n* = 37	Reference value	INI	OP	3 M	6 M	9 M	12 M	*post-hoc*
BMI [kg/m^2^]	18.5–24.9	42 ± 5.1	38 ± 4.5	32 ± 3.9	30 ± 3.5	29 ± 3.5	29 ± 3.7	a, b, c, d, e
BW [kg]	-	120 ± 17	109 ± 15	92 ± 14	86 ± 12	82 ± 13	82 ± 13	a, b, c, d, e
TWL [%]	-	ND	9.3 ± 4.1	16 ± 4.9	23 ± 4.8	28 ± 5.7	31 ± 7.2	b, c, d, e
T-CH [mg/dl]	115–190	192 ± 31	166 ± 35	181 ± 31	183 ± 40	189 ± 33	187 ± 23	a, b, c, d, e
LDL-CH [mg/dl]	<100	133 ± 29	98 ± 30	119 ± 31	114 ± 38	117 ± 30	111 ± 23	a, c, d
HDL-CH [mg/dl]	>40	48 ± 8.8	45 ± 7.2	49 ± 8.6	53 ± 10	57 ± 11	62 ± 11	a, b, c, d, e
TG [mg/dl]	<150	121 ± 33	131 ± 41	93 ± 30	88 ± 32	89 ± 46	83 ± 30	b, c, d, e
CRP [mg/l]	0.0–5.0	6.7 ± 5.9	7.0 ± 5.7	3.5 ± 3.6	3.2 ± 3.9	2.1 ± 2.2	1.9 ± 2.5	b, c, d, e
Glucose [mg/dl]	70–99	96 ± 11	95 ± 22	89 ± 10	87 ± 8.7	87 ± 7.8	86 ± 8.6	b, c, d, e
Insulin [µIU/ml]	2.0–25	15 ± 9.0	11 ± 7.7	7.8 ± 3.9	5.9 ± 2.8	6.4 ± 3.9	5.9 ± 3.3	b, c, d, e
HBA1C [mmol/mol]	<39	37 ± 3.2	ND	35 ± 2.5	34 ± 2.5	35 ± 2.2	35 ± 3.0	f, g, h, i
HOMA-IR	<2.5	3.6 ± 2.6	2.2 ± 1.4	1.8 ± 1.0	1.3 ± 0.7	1.4 ± 0.9	1.3 ± 0.8	a, c, d, e
GLP-1 agonist use (n)		16	16	0	0	0	0	
Antidepressant drug use (n)		17	17	17	17	17	17	
Antidiabetic drug use (n)		16	16	16	16	16	16	

Repeated Measures ANOVA with Bonferroni *post-hoc* test. In *post-hoc* test, significant (*p* < 0.05) are comparisons vs OP (or vs INI in HbA1c): a – INI vs OP, b – 3 M vs OP, c – 6 M vs OP, d – 9 M vs OP, e – 12 M vs OP.

*ND* no data obtained, *BMI* body mass index, *BW* body weight, *TWL* total weight loss, *T-CH* total cholesterol, *LDL-CH* low-density lipoprotein cholesterol, *HDL-CH* high-density lipoprotein cholesterol, *TG* triacylglycerols, *CRP* C-reactive protein, *HBA1C* glycated haemoglobin, *HOMA-IR* homoeostatic model assessment for insulin resistance, *INI* initial timepoint, *OP* operation timepoint.

### Diet and questionnaires

A summary of the dietary data is presented in Table 2. Caloric intake at the INI was significantly higher than at all postoperative time points. The lowest caloric intake was observed at 3 M post-SG, followed by a gradual increase over time; however, values at 12 M remained significantly lower than at baseline. Similar trends were noted for carbohydrate, fibre, and fat intake, which were lowest at 3 M post-SG and subsequently increased, though they remained significantly reduced at 12 M compared to baseline. Additionally, total protein intake significantly decreased at 3 M, 6 M, and 9 M post-SG compared to INI (*p* = 0.007, *p* = 0.005, *p* = 0.006, respectively). However, protein intake per kilogram of body weight increased over the course of treatment. Finally, the intake of branched-chain amino acids (BCAAs), which serve as substrates for branched-chain SCFAs (BSCFAs), [7] was analyzed. Although a decreasing trend was observed, no differences reached statistical significance in the *post-hoc* tests.

**Table 2:** Characteristics of diet during the study period based on 3-day food diaries (mean ± SD).

	INI	OP	3 M	6 M	9 M	12 M	*post-hoc*
Energy [kcal (MJ)]	1800 ± 583.7 (7.53 ± 2.44)	1222 ± 407.7 (5.12 ± 1.71)	1092 ± 273.6 (4.57 ± 1.14)	1183 ± 290.4 (4.95 ± 1.22)	1299.9 ± 322.8 (5.44 ± 1.35)	1440.9 ± 373.4 (6.03 ± 1.56)	a, e
Protein [g]	95.2 ± 33.4	90.3 ± 27.9	78.1 ± 25.1	77.9 ± 21.5	78.0 ± 21.5	84.9 ± 27.1	b, c, d
Protein for body mass [g/kg]	0.81 ± 0.27	0.86 ± 0.29	0.87 ± 0.32	0.95 ± 0.33	0.99 ± 0.35	1.09 ± 0.43	d, e
Carbohydrates [g]	205 ± 81.8	123 ± 64.5	112.3 ± 38.6	118 ± 37.5	138 ± 42.2	151 ± 46.9	a, e
Fats [g]	68.5 ± 26.4	43.8 ± 17.6	38.8 ± 14.6	46.1 ± 14.6	50.5 ± 16.8	56.8 ± 20.9	a, e
Fibre [g]	22.7 ± 8.2	17.7 ± 7.7	13.6 ± 6.4	14.1 ± 6.1	15.0 ± 4.8	15.7 ± 5.3	a, b, c, d
BCAA [g]	15.9 ± 6.6	15.7 ± 5.3	13.5 ± 5.3	13.3 ± 4.6	13.4 ± 4.4	13.9 ± 4.7	

Repeated Measures ANOVA with Bonferroni *post-hoc* test. In test, significant (*p* < 0.05) are comparisons vs OP: a – INI vs OP, b – 3 M vs OP, c – 6 M vs OP, d – 9 M vs OP, e – 12 M vs OP.

*BCAA* branched-chain amino acids, *INI* initial timepoint, *OP* operation timepoint.

To assess eating disorders, results from the EAT-26 questionnaire were compared. In contrast to other parameters, EAT-26 scores were compared directly to the INI phase (omitting the OP phase to avoid dietary bias), revealing significant improvements at 9 M and 12 M compared to INI (*p* = 0.044 and *p* = 0.034, respectively), indicating a positive shift in eating habits. Regarding the mental state, BDI-II scores showed significant differences between INI and all postoperative time points, suggesting a gradual and significant enhancement in patients’ mood after BS. The results are presented in Table 3.

**Table 3:** Result of Eating Attitudes Tests (EAT-26) and Beck’s Depression Inventory questionnaires (mean ± SD or median).

	INI	OP	3 M	6 M	9 M	12 M	*post-hoc*
EAT-26	14.5 ± 7.2	nd	15.9 ± 6.7	13.0 ± 6.3	11.1 ± 7.1	10.9 ± 6.3	h, i
BDI-II	15.6 ± 9.4	13.8 ± 8.8	9.6 ± 7.2	10.5 ± 8.5	7.6 ± 7.0	6.3 ± 6.8	b, c, d, e

Repeated Measures ANOVA with Bonferroni *post-hoc* test. In *post-hoc* test, significant (*p* < 0.05) are comparisons vs OP in BDI-II: b – 3 M vs OP, c – 6 M vs OP, d – 9 M vs OP, e – 12 M vs OP; and vs INI in EAT-26: h – 9 M vs INI; i – 12 M vs INI.

*EAT*-26 Eating Attitudes Test, *BDI*-II Beck’s Depression Inventory, *INI* initial timepoint, *OP* operation timepoint.

### SCFA concentrations in stool during treatment

No significant differences were found in SCFA profiles between the INI and OP time points. Among the major straight-chain SCFAs (SSCFAs), C2 concentrations were significantly reduced at 6 M, 9 M and 12 M post-SG compared to the OP point (*p* = 0.013, *p* = 0.015, and *p* = 0.046, respectively). Similarly, C3 was significantly decreased at 6 M (*p* = 0.012), 9 M (*p* = 0.012) and 12 M (*p* = 0.027) compared to OP. The concentration of C4 was lower at all postoperative time points compared to OP (*p* < 0.001 for all comparisons). Throughout the study, pentanoic acid (C5) showed a downward trend, whereas the levels of hexanoic acid (C6) and heptanoic acid (C7) showed an upward trend. Both total SCFA concentration and the combined concentration of SSCFAs showed a decreasing trend following SG. Significant differences were observed in the concentrations of individual BSCFAs after SG compared to OP. Notably, a downward trend was observed in 2-methylbutanoic acid (2-M-C4) concentration after surgery, though this did not reach statistical significance in the *post-hoc* test. In contrast, the level of 4-M-C5 was significantly higher at 6 M and 9 M after the procedure (*p* < 0.001 and *p* = 0.022). The faecal SCFA concentrations are shown in Table 4.

**Table 4:** The concentration of faecal short-chain fatty acids (SCFAs) in patients at the following time points before and after SG (median and 0.25 and 0.75 quartiles).

µmol/g of wet weight	INI	OP	3 M	6 M	9 M	12 M	*post-hoc*
Acetic acid	38.2 (23.0; 102)	32.1 (16.6; 115)	30.0 (14.1; 107)	27.5 (14.1; 107)	30.2 (15.8; 71.2)	28.4 (13.6; 84.2)	c, d, e
Propionic acid	21.6 (4.05; 34.0)	19.4 (6.80; 34.6)	17.2 (7.08; 25.9)	14.1 (10.6; 29.1)	13.7 (4.84; 37.7)	14.0 (6.49; 24.2)	a, c, d, e
Butyric acid	15.4 (8.48; 24.2)	15.2 (5.03; 26.6)	12.1 (9.50; 22.4)	11.9 (4.07; 17.1)	14.8 (7.64; 18.9)	15.3 (11.8; 18.4)	a, b, c, d, e
Pentanoic acid	1.73 (0.51; 2.31)	1.08 (0.76; 2.31)	1.25 (0.41; 3.16)	1.11 (0.75; 1.69)	1.53 (0.70; 2.49)	1.17 (0.69; 1.50)	a
Hexanoic acid	0.38 (0.29; 1.43)	0.39 (0.19; 1.01)	0.40 (0.23; 1.28)	0.42 (0.19; 0.75)	0.50 (0.12; 1.00)	0.72 (0.30; 1.39)	-
Heptanoic acid	0.06 (0.03; 0.17)	0.06 (0.03; 0.17)	0.05 (0.02; 0.15)	0.06 (0.03; 0.18)	0.09 (0.03; 0.31)	0.11 (0.03; 0.45)	-
2-methylbutanoic acid	0.21 (0.06; 0.39)	0.32 (0.09; 0.51)	0.65 (0.38; 1.02)	0.41 (0.28; 0.82)	0.46 (0.23; 0.69)	0.28 (0.15; 0.43)	NS
3-methylbutanoic acid	0.42 (0.09; 0.69)	0.41 (0.12; 0.70)	0.89 (0.38; 1.61)	0.72 (0.32; 1.25)	0.81 (0.41; 1.37)	0.50 (0.45; 0.95)	-
4-methylpentanoic acid	0.04 (0.02; 0.06)	0.04 (0.01; 0.08)	0.04 (0.01; 0.09)	0.09 (0.04; 0.46)	0.08 (0.03; 0.21)	0.08 (0.04; 0.13)	c, d
Total SCFAs	59.1 (31.1; 98.9)	55.3 (27.3; 155)	57.9 (42.2; 114.2)	56.3 (34.0; 82.8)	52.5 (35.1; 88.0)	47.6 (37.1; 89.6)	NS
Total straight SCFAs	58.9 (30.8; 98.6)	54.9 (27.2; 154.6)	55.3 (40.6; 114)	54.1 (33.3; 80.2)	50.4 (33.6; 85.7)	45.7 (34.4; 89.0)	-
Total branched SCFAs	0.49 (0.09; 1.07)	0.40 (0.08; 1.00)	1.35 (0.50; 2.47)	1.08 (0.74; 1.97)	1.23 (0.60; 2.13)	0.84 (0.50; 1.47)	-

Repeated Measures ANOVA with Bonferroni *post-hoc* test. Total straight SCFAs are the sum of acetic, propionic, butyric, pentanoic, hexanoic and heptanoic acids. Total branched SCFAs are the sum of 2-methylbutanoic, 3-methylbutanoic and 4-methylpentanoic acids. Total SCFAs are the sum of all mentioned SCFAs. In *post-hoc* test, significant (*p* < 0.05) are comparisons vs OP: a – INI vs OP, b – 3 M vs OP, c – 6 M vs OP, d – 9 M vs OP, e – 12 M vs OP.

*NS* not significant, *INI* initial timepoint, *OP* operation timepoint.

### Influence of comorbidities and medications on faecal BSCFA concentration

**Fig. 2:**
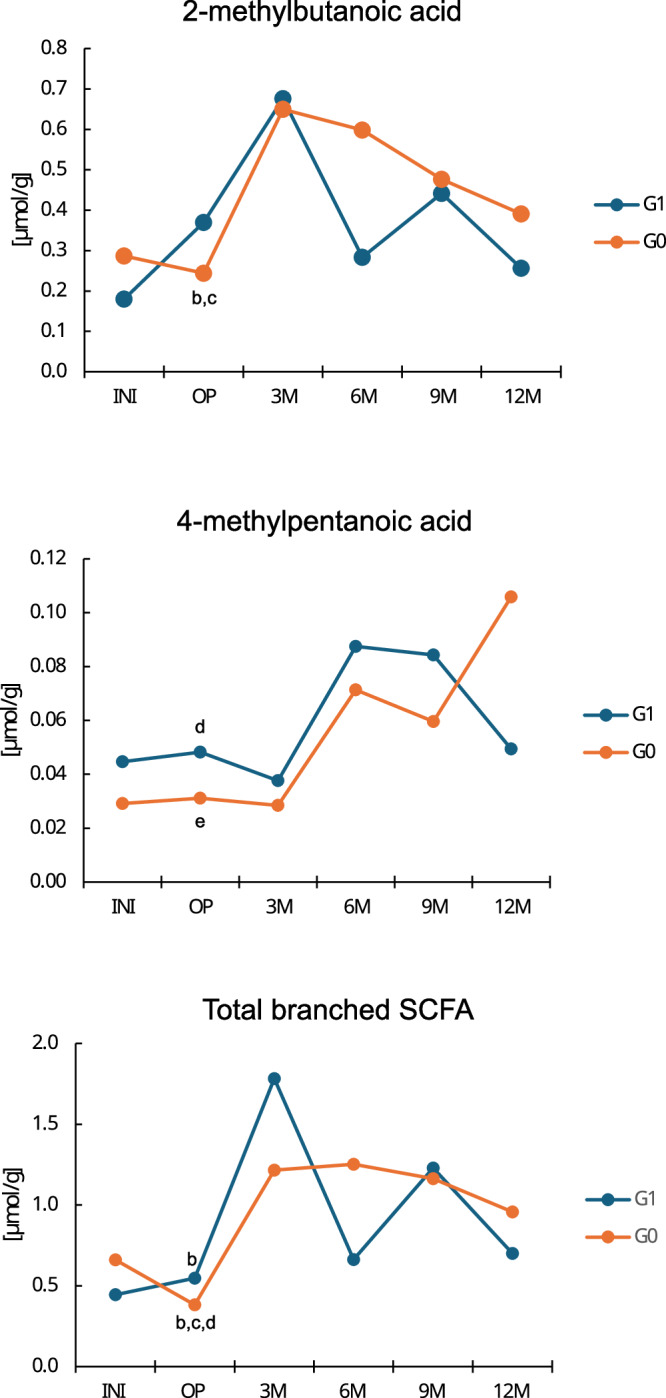
Comparison of changes in branched SCFA profiles in the group not using (G0) and using GLP-1 analogues (G1). Total branched SCFAs are the sum of 2-methylbutanoic, 3-methylbutanoic and 4-methylpentanoic acids. Presented are medians. Repeated Measures ANOVA with Bonferroni *post-hoc* test. In *post-hoc* tests, significant (*p* < 0.05) are comparisons vs OP: b – 3 M vs OP, c – 6 M vs OP, d – 9 M vs OP, e– 12 M vs OP.

Considering the strong association between obesity and various comorbidities and, consequently, different types of pharmacological treatment, we investigated whether these factors and the medication used might affect the changes in the SCFA profile induced by BS. Given the increasing focus on pharmacological weight-loss interventions, we specifically examined the role of GLP-1 analogues. A comparison of faecal SCFA profiles at specific time points following SG between patients who used liraglutide before surgery and those who did not (G1; *n* = 16 and G0; *n* = 21, respectively) revealed no significant differences in most parameters. However, a slight but significant increase in C5 level was observed in the G0 group at 12 M post-SG compared to OP. Furthermore, we analysed temporal changes within these two patient groups. Post-SG alterations in the 2-M-C4 and 4-M-C5 concentrations were observed in both cohorts; however, these elevations persisted longer in the G0 group. Specifically, 2-M-C4 levels remained elevated up to 6 M post-SG (*p* = 0.002), while 4-M-C5 levels remained high to 12 M (*p* = 0.028), compared to OP. Total BSCFA levels were elevated in G1 at 3 M (*p* = 0.003), whereas in G0, they remained elevated at 3 M, 6 M, and 9 M (*p* = 0.006, *p* = 0.002, and *p* = 0.048, respectively) (Fig. 2). Details are provided in Supplementary Table S2.

**Fig. 3:**
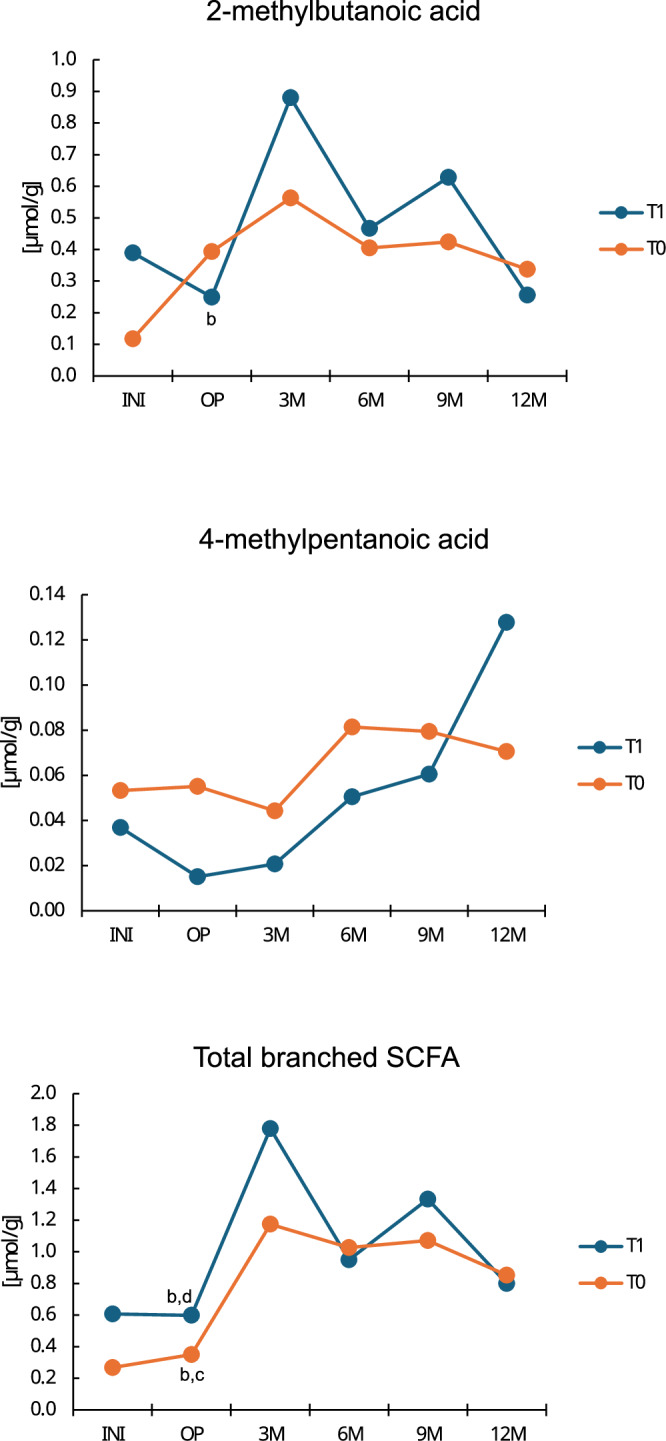
Comparison of changes in branched SCFA profiles in the group without (T0) and with diabetes or prediabetes (T1). Total branched SCFAs are the sum of 2-methylbutanoic, 3-methylbutanoic and 4-methylpentanoic acids. Presented are medians. Repeated Measures ANOVA with Bonferroni *post-hoc* test. In *post-hoc* tests, significant (*p* < 0.05) are comparisons vs OP: b – 3 M vs OP, c – 6 M vs OP, d – 9 M vs OP.

We have also investigated the differences in SCFA profiles between patients with and without diabetes or prediabetes (T1; *n* = 16 and T0; *n* = 21, respectively) diagnosed before treatment. While no significant differences in SCFA concentrations were found between the groups throughout the postoperative period, the patterns of change following BS differed. The T0 group exhibited modest changes, with a significant increase in total BSCFA levels only at 3 M and 6 M post-procedure compared to OP (*p* = 0.006 and *p* = 0.046). In contrast, the T1 group demonstrated more pronounced changes, including an increase in both 2-M-C4 and total BSCFA levels at 3 M post-SG (*p* = 0.014 and *p* = 0.003, respectively), and a significant elevation in total BSCFA levels at 9 M post-SG (*p* = 0.043) (Fig. 3). Details of these results are provided in Supplementary Table S3.

**Fig. 4:**
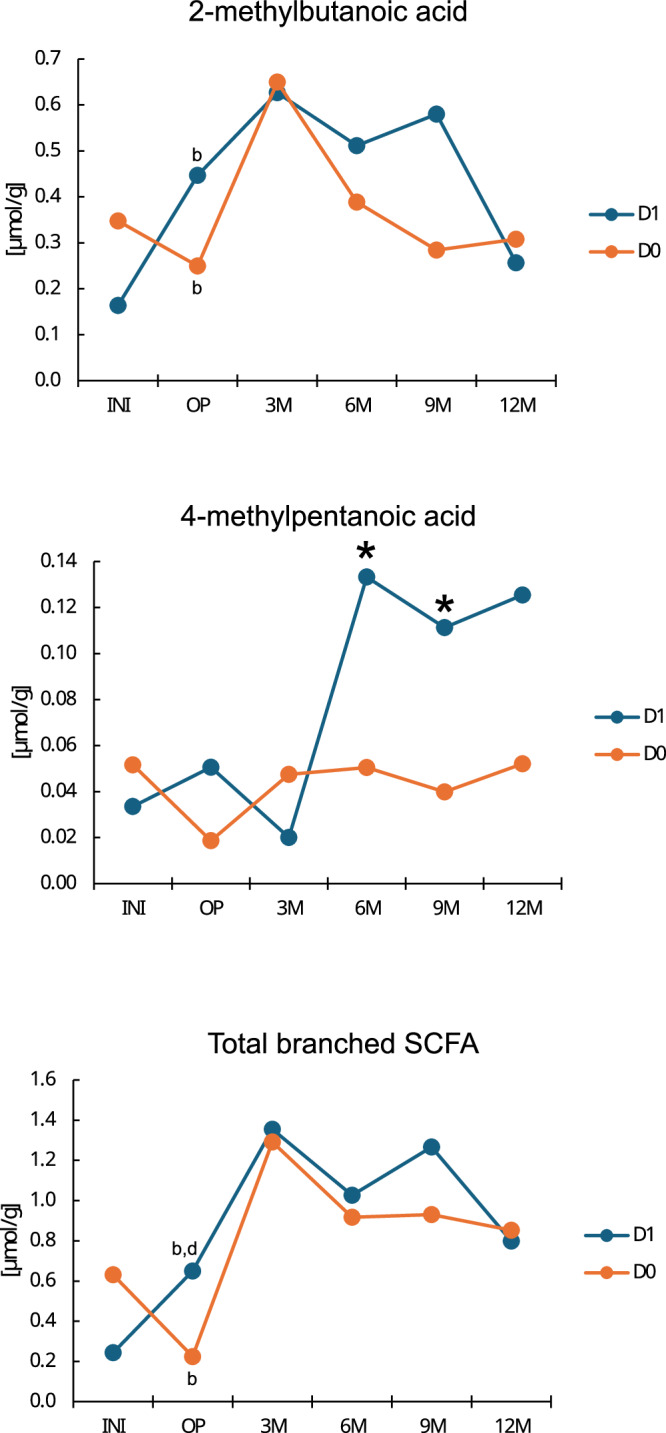
Comparison of changes in branched SCFA profiles in the group without (D0) and with depression (D1). Total branched SCFAs are the sum of 2-methylbutanoic, 3-methylbutanoic and 4-methylpentanoic acids. Presented are medians. Repeated Measures ANOVA with Bonferroni *post-hoc* test. In *post-hoc* tests, significant (*p* < 0.05) are comparisons vs OP: b – 3 M vs OP, d – 9 M vs OP. Comparison between groups with the Mann-Whitney U test, *p < 0.05.

Given the strong association between obesity and the prevalence of depressive disorders, we analysed the influence of depression and antidepressant medications on SCFA profiles. Comparing patients with depression (D1; *n* = 17) to those without (D0; *n* = 20), the D1 group showed a slight increase in C6 at OP (*p* = 0.014) and a slight decrease in C3 at 3 M after SG (*p* = 0.040). Moreover, the D1 cohort exhibited elevated concentrations of 4-M-C5 at both 6 M and 9 M compared to D0 (*p* = 0.031 and *p* < 0.001, respectively). In both D1 and D0, a significant increase in 2M-C4 and total BSCFA was observed at 3 M post-SG compared to OP (Fig. 4). The findings are summarised in Supplementary Table S4.

## Discussion

The most significant and novel finding of this study is the sustained increase in faecal BSCFAs at 9 and 12 months following LSG. Unlike the typical decline observed in major SCFAs, this BSCFA trajectory appears to be a distinct metabolic signature of post-SG recovery. Importantly, our analysis shows that this shift remains remarkably consistent regardless of patients’ diabetes status, presence of depression, or use of GLP-1 analogues, which have been linked with alterations in the gut microbiome [15–17]. This suggests that the physiological impact of SG on microbial protein fermentation is a robust phenomenon that may override pre-existing metabolic and psychological co-factors, potentially serving as a key driver for long-term clinical outcomes.

Diet is often described as a modulator of SCFA levels in faeces [18]. Despite a mandatory ‘liver-shrinking diet,’ with lower carbohydrate content, no significant differences were observed in the SCFA profiles in faecal samples during this period, likely due to its short duration (approximately 2 weeks).

BS leads to significant changes in the gut microbiota and their metabolites, profoundly impacting metabolic health [19]. Post-operatively, the substantial decline in the major SCFA levels in faeces aligns with data in the existing literature, including SG, as well as other types of BS such as Roux-en-Y gastric bypass (RYGB) or biliopancreatic diversion (BD) [11, 12, 19, 20]. This decline is often attributed to reduced energy harvesting and lower dietary fibre intake. In our cohort, fibre consumption consistently remained below the recommended intake (≥25 g/day) [21], so some further correction of diet may be considered to avoid metabolic consequences of inadequate fibre intake. Integrating targeted nutritional support is essential, as a 6-year follow-up has shown that chronic deficiencies in micronutrients and vitamins (e.g., iron, vitamin B12) remain a significant challenge after LSG [22]. Expanding supplementation to include probiotics and prebiotics may enhance the gut-derived metabolic effects of BS and restore SCFA levels, improving post-operative outcomes. Several randomised controlled trials have assessed the use of probiotics, including SCFA-producing strains from the *Lactobacillus* and *Bifidobacterium* genera, reporting short-term improvements in gastrointestinal symptoms, lipid metabolism, and liver function [23, 24]. Similarly, supplementation with specific prebiotics such as inulin, arabinogalactan, and resistant starch has been shown to significantly increase faecal SCFA concentrations and improve markers of metabolic health, including glycaemic control and lipid profile [25, 26].

The increase in BSCFAs, fermentation products of BCAA, is a noteworthy and novel finding. Although BSCFAs are markers of protein catabolism in the gut; we observed no correlation between their levels and BCAA intake, suggesting these alterations reflect shifts in gut microbial fermentation rather than diet [27, 28]. This may be linked to changes in specific taxa, particularly within the *Bacteroidetes* phylum, and the alteration in the *Firmicutes*/*Bacteroidetes* ratio previously described in this group of patients [7]. While the physiological role of BSCFAs is still being elucidated, they have been reported to modulate glucose metabolism and potentially improve insulin sensitivity [29, 30]. BSCFAs may also serve as an alternative energy source for colonocytes in cases of butyrate deficiency [31]. Notably, in our study, the BSCFA increase was more pronounced in patients with pre-existing diabetes or prediabetes, suggesting a protective role in glucose metabolism through improved insulin sensitivity and lipid handling [29], as also indicated by the *MILES* study [32]. While some reports suggest divergent metabolic trajectories for different surgical techniques, long-term evidence from a 10-year follow-up shows no significant differences between LSG, RYGB, and BD regarding diabetes resolution [33]. This suggests that despite anatomical differences, these procedures may result in similar long-term metabolic benefits, with the robust increase in BSCFAs identified in our study potentially acting as a common driver of these outcomes.

The collected data from BDI-II and EAT26 scores indicate enhanced emotional well-being and more mindful eating habits. Furthermore, existing evidence links BS with sustained reduction in anxiety and depressive symptoms 24 months after surgery [34]. However, the lack of strong correlation between SCFAs and mental health scores in our study, in contrast to the findings of Szczesniak et al. [35] suggests that the “gut-brain axis” response in bariatric patients is complex and may require longer observation to be fully understood.

The observed consistency in SCFA changes also reflects the overall clinical stability of the LSG procedure. While post-operative staple line leaks remain a rare but serious complication, recent evidence indicates that non-operative management, including percutaneous drainage and stenting, achieves high success rates and efficient resolution [36]. The fact that our cohort exhibited consistent metabolic changes, despite the inherent (though rare) surgical risks and diverse patient profiles, further highlights LSG as a robust intervention for metabolic remodelling.

Our study provides several important observations; however, it has some limitations, including a relatively small, all-female sample and reliance on self-reported questionnaires. Although the 12-month follow-up period provides valuable insights, long-term studies are required to assess the sustainability of observed alterations. Furthermore, the composition of the gut microbiota was not directly analysed, and conclusions about bacterial populations were inferred. Future research should incorporate metagenomic sequencing to determine whether BSCFA shifts correlate with specific bacterial expansions (e.g., *Bacteroidetes*).

In conclusion, while LSG induces broad remodelling of the gut environment, the prolonged elevation of BSCFAs appears to be a unique response of the post-BS microbiome. Our data show that the BSCFA surge is a consistent outcome of LSG, persisting across diverse patient profiles, including pre-diabetes, depression, and GLP-1 treatment. This consistency underscores the potency of surgical intervention in modulating gut-derived metabolites. Future studies integrating metagenomic and metabolomic approaches may help clarify the links between the gut microbiota, its metabolism, and the improvements observed after BS, and whether these metabolites could serve as biomarkers for surgical success or as targets for personalised post-BS nutritional support.

## Supplementary information

Supplementary material

## Data Availability

Raw data of study results have been uploaded to the following link: https://ppm.gumed.edu.pl/info/researchdata/GUM7fda2c458b3845049512976be623c170/.
