# Supplementary material for: Longitudinal analysis of short-chain fatty acid profiles in stool of sleeve gastrectomy patients
Source: Nutr Diabetes. 2026 Jun 11;16:24. doi: 10.1038/s41387-026-00441-x (PMC13396655; doi:10.1038/s41387-026-00441-x)
Supplement: Supplementary file 1 — Supplementary material [file 41387_2026_441_MOESM1_ESM.docx]

**Longitudinal analysis of short-chain fatty acid profiles in stool of sleeve gastrectomy patients**

**Supplemental information**

Supplementary Table S1. Target and confirmative ions of SCFA used in SIM mode.

| Short-chain fatty acid | Target ion (m/z) | Confirmative ion (m/z) |
| --- | --- | --- |
| Acetic acid | 117 | 75, 117 |
| Propionic acid | 131 | 75, 131 |
| Butyric acid | 145 | 75, 117, 145 |
| 2-methylbutyric acid | 159 | 75, 117, 159 |
| 3-methylbutyric acid | 159 | 75, 117, 159 |
| Pentanoic acid | 159 | 75, 117, 159 |
| 4-methylbutyric acid | 173 | 75, 117, 173 |
| Hexanoic acid | 173 | 75, 117, 173 |
| Heptanoic acid | 187 | 75, 117, 187 |

Supplementary Table S2. The concentration of faecal short-chain fatty acids (SCFAs) in GLP-1 patients and patients without using GLP-1 analogues at the following time points after LSG (median and 0.25 and 0.75 quartiles).

|  | No GLP-1 group (n=21) | | | | | | | GLP-1 group (n=16) | | | | | | |
| --- | --- | --- | --- | --- | --- | --- | --- | --- | --- | --- | --- | --- | --- | --- |
| µmol/g of wet weight | INI | OP | 3M | 6M | 9M | 12M | *post-hoc* | INI | OP | 3M | 6M | 9M | 12M | *post-hoc* |
| Acetic acid | 43.7 (28.0; 93.2) | 58.2 (16.6; 127) | 20.7 (11.1; 87.0) | 20.6 (14.3; 58.9) | 23.3 (15.6; 39.3) | 14.9 (9.45; 50.3) |  | 30.2 (19.6; 137) | 24.9 (16.6; 63.6) | 38.6 (15.9; 140) | 31.6 (15.1; 43.1) | 40.7 (20.4; 124) | 45.7 (20.1; 116) |  |
| Propionic acid | 21.6 (8.58; 31.2) | 25.6 (6.99; 50.8) | 17.6 (7.81; 24.1) | 20.1 (10.8; 33.0) | 12.4 (4.95; 23.2) | 12.2 (7.79; 25.5) |  | 22.9 (3.49; 34.1) | 17.0 (6.89; 25.4) | 15.1 (8.25; 34.4) | 13.2 (10.4; 23.8) | 29.4 (5.09; 45.7) | 16.1 (6.66; 21.9) |  |
| Butyric acid | 16.6 (8.48; 24.6) | 13.5 (3.38; 27.4) | 12.4 (9.98; 21.9) | 6.20 (2.86; 17.8) | 14.9 (8.41; 19.1) | 14.7 (11.6; 18.1) |  | 15.4 (9.32; 20.6) | 15.1 (7.29; 25.2) | 11.1 (7.24; 24.4) | 13.3 (9.86; 14.6) | 14.3 (7.18; 18.8) | 14.8 (11.9; 15.9) |  |
| Pentanoic acid | 1.76 (0.42; 2.83) | 0.87 (0.55; 1.93) | 0.88 (0.37; 2.50) | 1.39 (0.66; 1.72) | 1.28 (0.49; 2.47) | 1.35 (1.10; 2.01) |  | 1.72 (0.73; 2.09) | 1.77 (0.92; 2.77) | 2.18 (0.73; 3.74) | 1.04 (0.87; 1.39) | 1.93 (0.84; 2.68) | 0.91 (0.50; 1.24) | NS |
| Hexanoic acid | 0.35 (0.29; 1.29) | 0.34 (0.18; 1.01) | 0.38 (0.17; 0.72) | 0.34 (0.17; 0.62) | 0.31 (0.12; 0.93) | 0.55 (0.26; 0.77) |  | 0.42 (0.27; 1.52) | 0.39 (0.22; 1.56) | 0.43 (0.35; 1.91) | 0.46 (0.33; 1.14) | 0.52 (0.21; 1.19) | 0.94 (0.54; 1.40) |  |
| Heptanoic acid | 0.08 (0.03; 0.17) | 0.06 (0.03; 0.21) | 0.03 (0.02; 0.15) | 0.08 (0.03; 0.18) | 0.09 (0.03; 0.25) | 0.07 (0.02; 0.42) |  | 0.05 (0.03; 0.14) | 0.06 (0.03; 0.12) | 0.08 (0.03; 0.17) | 0.04 (0.03; 0.18) | 0.17 (0.02; 0.31) | 0.14 (0.05; 0.57) |  |
| 2-methylbutanoic acid | 0.29 (0.04; 0.39) | 0.24 (0.05; 0.54) | 0.65 (0.40; 0.95) | 0.60 (0.39; 0.99) | 0.48 (0.23; 0.63) | 0.39 (0.23; 0.44) | c, c | 0.18 (0.10; 0.39) | 0.37 (0.27; 0.51) | 0.68 (0.35; 1.02) | 0.28 (0.21; 0.47) | 0.44 (0.28; 0.72) | 0.26 (0.15; 0.36) | NS |
| 3-methylbutanoic acid | 0.46 (0.07; 0.72) | 0.88 (0.41; 1.58) | 0.88 (0.42; 1.75) | 0.76 (0.40; 1.33) | 0.84 (0.37; 1.35) | 0.52 (0.45; 0.74) |  | 0.32 (0.18; 0.62) | 1.09 (0.39; 1.57) | 1.09 (0.29; 1.57) | 0.50 (0.32; 0.85) | 0.79 (0.42; 1.24) | 0.49 (0.44; 1.07) |  |
| 4-methylpentanoic acid | 0.03 (0.02; 0.06) | 0.03 (0.01; 0.08) | 0.03 (0.01; 0.09) | 0.07 (0.03; 0.13) | 0.06 (0.03; 0.10) | 0.11 (0.05; 0.16) | e | 0.04 (0.03; 0.06) | 0.05 (0.02; 0.07) | 0.04 (0.01; 0.09) | 0.09 (0.03; 0.40) | 0.08 (0.03; 0.45) | 0.05 (0.04; 0.12) | d |
| Total SCFAs | 65.5 (33.2; 98.0) | 60.5 (28.8; 171) | 78.2 (42.2; 124) | 55.8 (29.0; 94.8) | 51.2 (34.2; 83.1) | 38.9 (22.0; 71.6) |  | 50.4 (30.9; 115) | 46.4 (25.1; 84.4) | 51.1 (39.5; 88.9) | 57.5 (41.9; 74.1) | 63.2 (37.6; 146) | 62.5 (43.7; 108) |  |
| Total straight SCFAs | 65.4 (32.4; 97.3) | 59.8 (28.8; 171) | 76.4 (40.6; 124) | 53.6 (26.5; 94.4) | 50.4 (32.6; 81.9) | 37.9 (21.3; 70.1) |  | 50.3 (30.9; 114) | 46.1 (25.0; 83.1) | 49.1 (38.6; 87.5) | 56.4 (41.2; 72.3) | 61.2 (36.6; 144) | 61.4 (43.1; 108) |  |
| Total branched SCFAs | 0.66 (0.09; 1.11) | 0.38 (0.11; 0.84) | 1.22 (0.54; 2.34) | 1.25 (0.76; 2.42) | 1.16 (0.61; 2.13) | 0.96 (0.73; 1.56) | b, c, d | 0.44 (0.09; 0.89) | 0.55 (0.08; 1.01) | 1.78 (0.52; 2.56) | 0.66 (0.44; 1.05) | 1.23 (0.60; 1.86) | 0.70 (0.42; 1.01) | b |

Repeated Measures ANOVA with Bonferroni *post-hoc* test. NS-not significant. Total straight SCFAs are the sum of acetic, propionic, butyric, pentanoic, hexanoic and heptanoic acids. Total branched SCFAs are the sum of 2-methylbutanoic, 3-methylbutanoic and 4-methylpentanoic acids. Total SCFAs are the sum of all mentioned SCFAs. INI – initial timepoint, OP – operation timepoint. In *post-hoc* test significant (p<0.05) are comparisons vs OP: b – 3M vs OP, c – 6M vs OP, d – 9M vs OP, e – 12M vs OP.

Supplementary Table S3. The concentration of faecal short-chain fatty acids (SCFAs) in patients with and without diabetes or prediabetes at the following time points after LSG (median and 0.25 and 0.75 quartiles).

|  | No diabetes group (n=21) | | | | | | | Diabetes group (n=16) | | | | | | |
| --- | --- | --- | --- | --- | --- | --- | --- | --- | --- | --- | --- | --- | --- | --- |
| µmol/g of wet weight | INI | OP | 3M | 6M | 9M | 12M | *post-hoc* | INI | OP | 3M | 6M | 9M | 12M | *post-hoc* |
| Acetic acid | 45.6 (22.6; 159) | 59.2 (18.5; 119) | 32.7 (10.2; 25.1) | 29.0 (14.2; 66.6) | 34.3 (13.6; 70.6) | 20.5 (9.45; 62.9) |  | 27.7 (23.0; 53.2) | 24.9 (13.1; 108) | 16.9 (9.46; 162) | 19.9 (14.4; 33.4) | 29.5 (17.7; 65.7) | 45.7 (21.1; 156) |  |
| Propionic acid | 25.4 (7.95; 40.1) | 19.3 (5.71; 38.7) | 19.4 (10.1; 25.1) | 13.8 (10.3; 36.1) | 10.8 (3.14; 44.6) | 11.3 (2.94; 20.6) |  | 10.3 (3.36; 29.7) | 19.8 (12.9; 29.4) | 13.3 (2.60; 24.4) | 18.8 (11.4; 23.7) | 14.0 (7.08; 289) | 16.4 (8.66; 28.9) |  |
| Butyric acid | 18.1 (10.0; 23.9) | 18.1 (4.64; 27.8) | 11.5 (9.49; 25.3) | 8.29 (3.11; 17.3) | 16.5 (8.88; 19.8) | 15.9 (11.9; 18.9) |  | 15.3 (11.2; 26.9) | 13.2 (9.68; 20.1) | 14.7 (11.1; 20.1) | 12.8 (6.61; 16.4) | 11.3 (6.57; 16.3) | 13.4 (10.8; 16.7) |  |
| Pentanoic acid | 1.65 (0.52; 2.19) | 1.05 (0.75; 2.24) | 1.22 (0.39; 2.74) | 1.02 (0.77; 1.74) | 1.56 (0.54; 2.43) | 1.32 (0.93; 1.58) |  | 1.80 (0.39; 3.12) | 1.08 (0.81; 2.68) | 1.28 (0.42; 3.75) | 1.38 (0.86; 1.39) | 1.49 (0.96; 2.69) | 0.69 (0.51; 1.34) |  |
| Hexanoic acid | 0.41 (0.30; 1.54) | 0.39 (0.17; 0.90) | 0.43 (0.23; 1.32) | 0.41 (0.20; 0.65) | 0.43 (0.13; 0.88) | 0.91 (0.27; 1.53) |  | 0.39 (0.25; 1.16) | 0.63 (0.23; 1.32) | 0.40 (0.28; 0.96) | 0.43 (0.23; 1.52) | 0.51 (0.12; 1.77) | 0.69 (0.43; 0.77) |  |
| Heptanoic acid | 0.07 (0.03; 0.17) | 0.06 (0.03; 0.16) | 0.05 (0.02; 0.15) | 0.06 (0.03; 0.18) | 0.09 (0.02; 0.24) | 0.06 (0.03; 0.63) |  | 0.06 (0.03; 0.16) | 0.06 (0.04; 0.17) | 0.05 (0.03; 0.17) | 0.06 (0.03; 0.18) | 0.13 (0.03; 0.41) | 0.14 (0.11; 0.31) |  |
| 2-methylbutanoic acid | 0.12 (0.04; 0.35) | 0.39 (0.08; 0.52) | 0.56 (0.36; 0.85) | 0.41 (0.24; 0.73) | 0.42 (0.20; 0.57) | 0.34 (0.18; 0.43) |  | 0.39 (0.20; 0.46) | 0.25 (0.14; 0.46) | 0.88 (0.43; 1.29) | 0.47 (0.34; 0.88) | 0.63 (0.26; 0.80) | 0.26 (0.15; 0.41) | b |
| 3-methylbutanoic acid | 0.23 (0.07; 0.72) | 0.62 (0.12; 0.74) | 0.89 (0.40; 1.57) | 0.73 (0.33; 1.23) | 0.79 (0.42; 1.07) | 0.51 (0.45; 0.75) |  | 0.46 (0.32; 0.63) | 0.33 (0.24; 0.44) | 0.89 (0.36; 1.71) | 0.68 (0.34; 1.19) | 0.84 (0.41; 1.44) | 0.47 (0.46; 1.02) |  |
| 4-methylpentanoic acid | 0.05 (0.02; 0.07) | 0.06 (0.02; 0.08) | 0.04 (0.02; 0.10) | 0.08 (0.02; 0.20) | 0.08 (0.03; 0.14) | 0.07 (0.04; 0.11) |  | 0.04 (0.02; 0.05) | 0.02 (0.01; 0.03) | 0.02 (0.01; 0.07) | 0.05 (0.04; 0.30) | 0.06 (0.03; 0.42) | 0.13 (0.05; 0.37) | NS |
| Total SCFAs | 71.4 (41.4; 182) | 60.5 (28.1; 156) | 73.4 (46.2; 126) | 54.9 (34.0; 87.3) | 51.1 (27.5; 92.7) | 40.7 (30.7; 85.8) |  | 39.4 (27.2; 73.8) | 46.8 (25.2; 88.9) | 46.8 (29.2; 89.0) | 58.7 (38.5; 72.4) | 52.5 (41.8; 73.8) | 62.8 (54.9; 87.1) |  |
| Total straight SCFAs | 70.3 (41.3; 181) | 59.8 (28.0; 156) | 71.4 (43.3; 126) | 53.2 (33.3; 86.3) | 50.7 (26.8; 90,3) | 40.2 (30.0; 84.5) |  | 39.1 (26.0; 73.0) | 46.4 (25.0; 88.5) | 45.4 (25.8; 87.9) | 57.6 (38.1; 70.1) | 50.4 (40.5; 72.0) | 62.0 (53.2; 86.4) |  |
| Total branched SCFAs | 0.27 (0.08; 0.89) | 0.35 (0.08; 1.28) | 1.17 (0.51; 2.28) | 1.03 (0.65; 1.83) | 1.07 (0.55; 1.70) | 0.85 (0.53; 1.43) | b, c | 0.61 (0.11; 1.07) | 0.60 (0.12; 0.73) | 1.78 (0.50; 2.60) | 0.95 (0.39; 1.99) | 1.33 (0.69; 2.15) | 0.80 (0.42; 1.47) | b, d |

Repeated Measures ANOVA with Bonferroni *post-hoc* test. NS-not significant. Total straight SCFAs are the sum of acetic, propionic, butyric, pentanoic, hexanoic and heptanoic acids. Total branched SCFAs are the sum of 2-methylbutanoic, 3-methylbutanoic and 4-methylpentanoic acids. Total SCFAs are the sum of all mentioned SCFAs. INI – initial timepoint, OP – operation timepoint. In *post-hoc* test significant (p<0.05) are comparisons vs OP: b – 3M vs OP, c – 6M vs OP, d – 9M vs OP.

Supplementary Table S4. The concentration of faecal short-chain fatty acids (SCFAs) in patients with and without depression at the following time points after LSG (median and 0.25 and 0.75 quartiles).

|  | No depression group (n=20) | | | | | | | Depression group (n=17) | | | | | | |
| --- | --- | --- | --- | --- | --- | --- | --- | --- | --- | --- | --- | --- | --- | --- |
| µmol/g of wet weight | INI | OP | 3M | 6M | 9M | 12M | *post-hoc* | INI | OP | 3M | 6M | 9M | 12M | *post-hoc* |
| Acetic acid | 67.7 (22.7; 159) | 32.8 (18.8; 92.9) | 41.8 (11.7; 124) | 20.6 (13.7; 53.1) | 29.5 (15.4; 52.6) | 38.6 (14.1; 107) |  | 28.3 (23.3; 62.8) | 36.2 (13.1; 128) | 24.7 (17.2; 80.2) | 29.9 (15.2; 44.6) | 31.2 (16.5; 75.5) | 21.3 (13.4; 54.7) |  |
| Propionic acid | 24.2 (3.19; 30.7) | 13.2 (4.09; 35.5) | 20.3 (12.1; 38.1) | 16.2 (10.0; 23.6) | 6.50 (3.25; 28.1) | 13.0 (4.09; 23.9) |  | 16.3 (6.26; 35.6) | 25.4 (17.0; 31.3) | 11.6 (7.01; 17.2) | 14.1 (10.7; 49.4) | 19.3 (6.15; 45.1) | 14.4 (8.66; 23.2) |  |
| Butyric acid | 16.7 (7.58; 24.4) | 11.7 (5.16; 19.5) | 14.9 (10.2; 23.6) | 8.29 (3.43; 17.8) | 16.2 (7.95; 28.1) | 15.1 (11.8; 17.7) |  | 15.3 (9.52; 23.7) | 24.5 (4.98; 30.5) | 10.9 (7.25; 18.2) | 13.7 (5.27; 15.7) | 13.7 (3.77; 17.8) | 16.5 (14.0; 19.1) |  |
| Pentanoic acid | 2.10 (1.17; 2.41) | 0.90 (0.66; 2.03) | 1.58 (0.71; 2.97) | 1.14 (0.71; 1.48) | 1.93 (0.75; 2.75) | 1.17 (0.73; 1.55) |  | 1.07 (0.31; 1.85) | 1.46 (0.94; 2.68) | 0.58 (0.37; 3.30) | 1.07 (0.91; 1.71) | 1.11 (0.71; 2.26) | 1.20 (0.63; 1.49) |  |
| Hexanoic acid | 0.31 (0.29; 1.43) | 0.20 (0.17; 0.43) | 0.50 (0.22; 1.34) | 0.36 (0.17; 0.65) | 0.24 (0.12; 0.96) | 0.80 (0.57; 1.37) |  | 0.82 (0.29; 1.47) | 0.97 (0.37; 2.27) | 0.40 (0.25; 0.74) | 0.46 (0.30; 1.22) | 0.51 (0.27; 0.95) | 0.23 (0.10; 1.04) |  |
| Heptanoic acid | 0.03 (0.09) | 0.05 (0.02; 0.12) | 0.04 (0.02; 0.19) | 0.06 (0.03; 0.20) | 0.06 (0.02; 0.28) | 0.12 (0.06; 0.47) |  | 0.12 (0.05; 0.18) | 0.06 (0.04; 0.18) | 0.06 (0.02; 0.13) | 0.06 (0.03; 0.17) | 0.09 (0.05; 0.28) | 0.03 (0.01; 0.29) |  |
| 2-methylbutanoic acid | 0.35 (0.12; 0.45) | 0.25 (0.07; 0.43) | 0.65 (0.35; 1.29) | 0.39 (0.28; 0.82) | 0.28 (0.23; 0.63) | 0.31 (0.17; 0.41) | b | 0.16 (0.05; 0.37) | 0.45 (0.19; 0.51) | 0.63 (0.44; 0.92) | 0.51 (0.27; 0.81) | 0.58 (0.42; 0.79) | 0.26 (0.15; 0.47) | b |
| 3-methylbutanoic acid | 0.60 (0.25; 0.71) | 0.41 (0.09; 0.66) | 0.89 (0.35; 1.65) | 0.72 (0.40; 1.04) | 0.58 (0.43; 1.31) | 0.51 (0.46; 0.88) |  | 0.22 (0.08; 0.63) | 0.47 (0.29; 0.76) | 0.89 (0.42; 1.15) | 0.67 (0.28; 1.29) | 0.83 (0.31; 1.35) | 0.47 (0.45; 0.98) |  |
| 4-methylpentanoic acid | 0.05 (0.02; 0.09) | 0.02 (0.01; 0.06) | 0.05 (0.02; 0.09) | 0.05 (0.02; 0.08) | 0.04 (0.03; 0.08) | 0.05 (0.04; 0.10) |  | 0.03 (0.02; 0.05) | 0.05 (0.02; 0.08) | 0.02 (0.01; 0.10) | 0.13 (0.05; 0.45) | 0.11 (0.03; 0.36) | 0.13 (0.08; 0.33) | NS |
| Total SCFAs | 62.5 (35.5; 140) | 52.9 (24.1; 99.6) | 88.9 (43.9; 119) | 53.2 (29.9; 74.9) | 47.7 (28.6; 85.1) | 39.4 (25.7; 71.3) |  | 59.1 (31.0; 93.4) | 65.0 (35.4; 169) | 47.7 (41.9; 94.4) | 58.6 (41.9; 87.7) | 63.6 (36.5; 122) | 62.8 (44.6; 96.2) |  |
| Total straight SCFAs | 61.9 (35.0; 140) | 52.5 (24.1; 99.0) | 87.6 (41.7; 118) | 52.5 (26.1; 74.6) | 47.6 (27.7; 84.2) | 38.4 (24.6; 68.4) |  | 58.9 (29.9; 92.1) | 64.1 (34.7; 169) | 46.2 (40.1; 93.7) | 56.4 (41.3; 85.3) | 61.7 (35.3; 121) | 62.0 (43.5; 95.8) |  |
| Total branched SCFAs | 0.63 (0.09; 1.12) | 0.22 (0.06; 0.77) | 1.29 (0.53; 2.51) | 0.92 (0.65; 1.42) | 0.93 (0.61; 1.95) | 0.85 (0.52; 1.27) | b | 0.24 (0.09; 0.70) | 0.65 (0.13; 1.06) | 1.35 (0.43; 1.96) | 1.03 (0.44; 2.10) | 1.27 (0.61; 2.11) | 0.80 (0.28; 1.64) | b, d |

Repeated Measures ANOVA with Bonferroni *post-hoc* test. NS-not significant. Total straight SCFAs are the sum of acetic, propionic, butyric, pentanoic, hexanoic and heptanoic acids. Total branched SCFAs are the sum of 2-methylbutanoic, 3-methylbutanoic and 4-methylpentanoic acids. Total SCFAs are the sum of all mentioned SCFAs. INI – initial timepoint, OP – operation timepoint. In *post-hoc* test significant (p<0.05) are comparisons vs OP: b – 3M vs OP, d – 9M vs OP.
